# Novel Eu^2+^-activated thiogallate phosphors for white LED applications: structural and spectroscopic analysis†

**DOI:** 10.1039/c8ra01113c

**Published:** 2018-03-27

**Authors:** Szu-Ping Lee, Ting-Shan Chan, Somrita Dutta, Teng-Ming Chen

**Affiliations:** Phosphors Research Laboratory, Department of Applied Chemistry, National Chiao Tung University Hsinchu 30010 Taiwan tmchen@mail.nctu.edu.tw; National Synchrotron Radiation Research Center Hsinchu 30076 Taiwan

## Abstract

Novel Eu^2+^-activated BaGa_2_SiS_6_ and Ba_2_Ga_8_SiS_16_ thiogallate phosphors were prepared by solid-state reaction route. The BaGa_2_SiS_6_:Eu^2+^ phosphor generated a green emission upon excitation at 405 nm, whereas the Ba_2_Ga_8_SiS_16_:*x*Eu^2+^ phosphor could be tuned from cyan to green range with increasing Eu^2+^ concentration upon excitation at 365 nm. Additionally, the thermal luminescence properties of the thiogallate phosphors were investigated in the temperature range of 25 to 250 °C. A warm-white LED is fabricated using the combination of a 405 nm blue InGaN-based LED chip with the green-emitting BaGa_2_SiS_6_:0.01Eu^2+^ phosphor, and red-emitting Sr_2_Si_5_N_8_:Eu^2+^ commercial phosphor with the CRI value of ∼88 and the CCT of 4213 K.

## Abstract 2

BaGa_2_SiS_6_:Eu^2+^ (a) emits a green light upon excitation at 405 nm, whereas Ba_2_Ga_8_SiS_16_:*x*Eu^2+^ (b) can be tuned to emit cyan to green with increasing Eu^2+^ content when excited at 365 nm.

## Graphical abstract

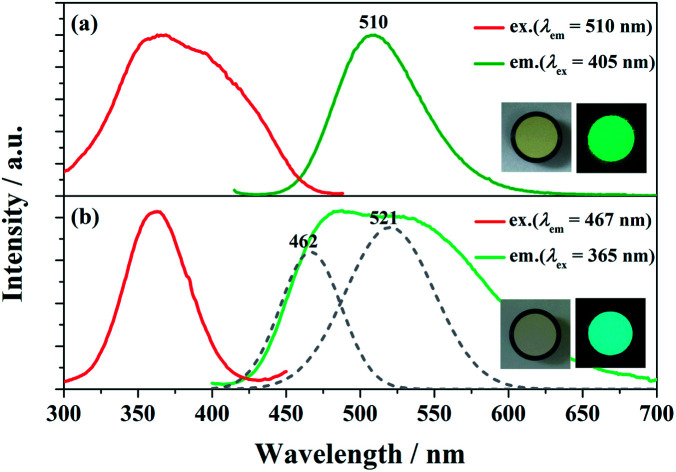

## 1. Introduction

Phosphor-converted white light emitting diodes (PC-WLEDs) have emerged as one of the most promising and eco-friendly white-light sources for general illumination, consuming less energy than conventional incandescent light sources^1–6^. In most commercial WLEDs, a combination of a blue LED chip and cerium doped yttrium aluminum garnet (YAG:Ce^3+^) phosphor is used for the generation of white light.^7^ But this combination has the shortcomings of poor colour rendering index (CRI) and a high correlated colour temperature (CCT) due to the absence of emission in the red spectral region which restricts its use in commercial lighting.^8,9^ There are several approaches considered for achieving high CRI and cool color correlated temperature such as; a combination of a blue LED chip with a yellow-emitting and a narrow-band red-emitting phosphor or a mixture of green-emitting and red-emitting phosphor.^8–12^ The use of a near-ultraviolet (n-UV) LED chip or an ultraviolet (UV) LED chip when combined with a mixture of red, green and blue phosphors can also improve the CRI value and the CCT values of the white light generated.^13–15^ Hence there is a need to develop new phosphors for obtaining the optimal requirements for a high-quality white light for general commercial lighting applications.

One of the main approaches involved in the designing of new phosphors includes the exploration of host compounds from existing structural model and then the proper selection of suitable activators (such as broadband emitting Eu^2+^, Ce^3+^, and Mn^2+^).^16^ Two such hosts that can be considered for exploration are BaGa_2_SiS_6_ reported by Yin *et al.* in 2012 (ref. 17) and Ba_2_Ga_8_SiS_16_ reported by Liu and group in the year 2014 (ref. 18) for the first time. Both the materials were studied for high power IR – nonlinear optical applications. Since, sulphide-containing lattices when activated by Eu^2+^ provide a longer emission wavelength due to their higher nephelauxetic effect compared to nitrides and oxides,^19–21^ the structural design of these two materials opens up the possibility to study them for LED applications. The gallium (Ga^3+^) and silicon (Si^4+^) ions can create a protective environment to successfully enhance the stability of the sulfide phosphors and hence their luminescence properties.

When doped with Eu^2+^, the emission spectrum is in general characterized by a single broad band emission which can be deconvoluted into two or more number of emission bands depending on the number of cationic sites replaced by Eu which can also help in achieving higher CRI values for WLED applications. Also, due to the small Stokes shift value, the phosphors could be excited from UV to blue region. The covalency and the crystal field splitting between the host allows Eu^2+^ ions to exhibit the parity-allowed 5d^1^4f^*n*−1^ → 4f^*n*^ emission from the UV region to the visible spectral region.^22–26^ In this research, a green-emitting Eu^2+^-activated BaGa_2_SiS_6_ and a tunable cyan-to-green-emitting Ba_2_Ga_8_SiS_16_:Eu^2+^ phosphors were reported which show broad excitation range. The structural and luminescent properties of the phosphors were investigated in detail. In addition, a LED device using the BaGa_2_SiS_6_:Eu^2+^ phosphor with a 405 nm LED chip was fabricated to demonstrate its applicability as a colour-conversion phosphor in the fabrication of WLEDs.

## 2. Experimental

### 2.1 Materials and synthesis

The Eu^2+^ -doped BaGa_2_SiS_6_ and Ba_2_Ga_8_SiS_16_ phosphors were synthesized using BaS (Alfa Aesar, 99.7%), Ga_2_S_3_ (Alfa Aesar, 99.99%), Si powder (Alfa Aesar, 99.999%), and S powder (Acros, 99.999%) and EuF_2_ (Alfa Aesar, 99.9%) as raw ingredients. The ingredients were homogeneously mixed and ground in a glove box under nitrogen atmosphere and loaded into the quartz glass ampoules, which were then sealed off after evacuated to 10^−4^ torr. The ampoules were heated in a furnace to 900 °C for 8 h at 5 °C min^−1^. Finally, the powdered phosphors were obtained after the furnace naturally cooled down to room temperature. In each case, the reactions are summarized in the following equations.

1 (1 − *x*)BaS + Ga_2_S_3_ + *x*EuF_2_ + Si + 2S → (Ba_1−*x*_Eu_*x*_)Ga_2_SiS_6_

2 (2 − 2*x*)BaS + 4Ga_2_S_3_ + 2*x*EuF_2_ + 2Si + 4S → (Ba_1−*x*_Eu_*x*_)_2_Ga_8_SiS_16_ + SiS_2_↑

### 2.2 Characterizations

Synchrotron X-ray Diffraction (SXRD) using the BL01C2 beamline with an X-ray wavelength of 0.774908 Å was used to analyse the phase purity of the synthesized products by at the National Synchrotron Radiation Research Centre (NSRRC) in Hsinchu, Taiwan. The X-ray Rietveld refinement was carried to investigate the structure of the phosphor using the General Structure Analysis System (GSAS) software. JEOL JSM-7401F operated at voltage of 5 kV was used to perform the scanning electron microscopy (SEM) morphological analysis and energy dispersive X-ray spectroscopy (EDS) analysis. The photoluminescence spectra and the time resolved measurement of the phosphors were obtained using a FS5 Fluorescence Spectrometer (Edinburgh Instruments) with a 450 W xenon lamp and a TCSPC (Time Correlated Single Photon Counting) module in combination with EPLED-360 picosecond pulsed light emitting diode laser system as the excitation source respectively. An integrating sphere whose inner face was coated with Spectralon equipped with a spectrofluorometer (Horiba Jobin-Yvon Fluorolog 3-2-2) measured the quantum efficiency (QE). The thermal luminescence performance was analyzed using a heating apparatus (THMS-600) fitted with PL equipment. The electroluminescence (EL) spectra were performed by Sphere-Optics integrating sphere with LED measurement starter packages (Onset, Inc.) recording at different current in the range of 100–300 mA.

## 3. Results and discussion

### 3.1 Structural characterizations and crystallographic parameters of the (Ba_0.95_Eu_0.05_)Ga_2_SiS_6_ and (Ba_0.90_Eu_0.10_)_2_Ga_8_SiS_16_ phosphors

**Fig. 1:**
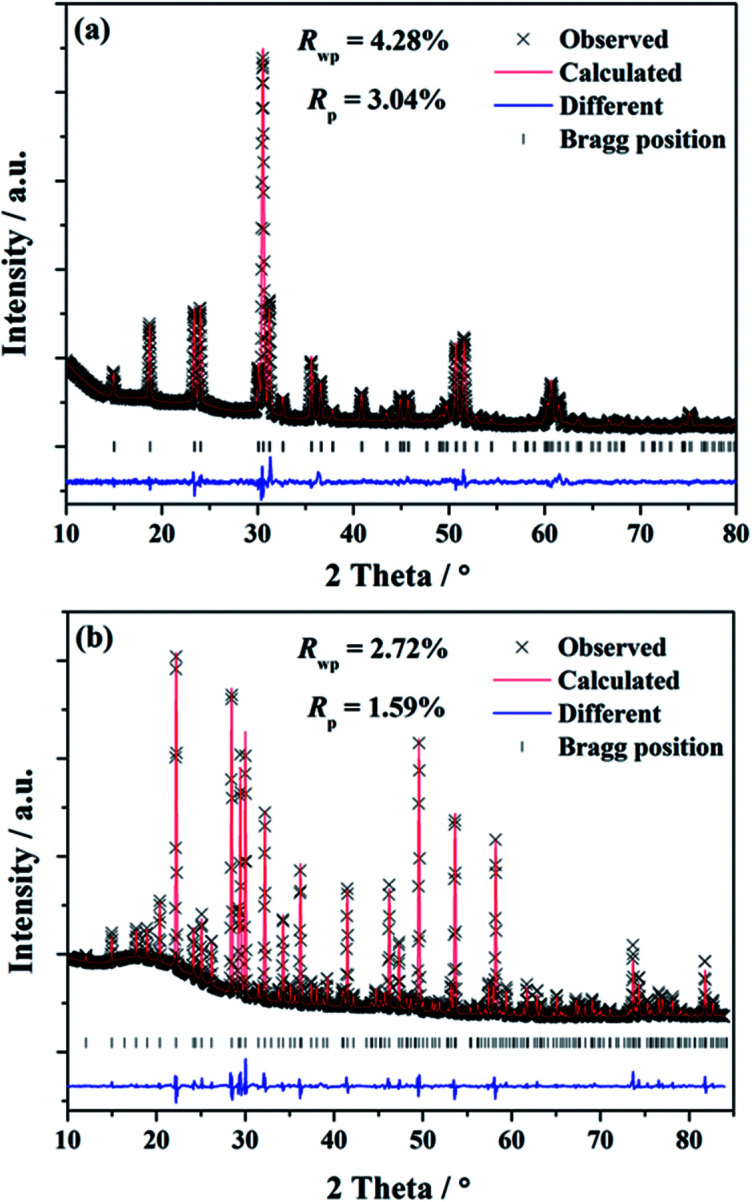
The SXRD profiles for Rietveld refinement results of (a) (Ba_0.95_Eu_0.05_)Ga_2_SiS_6_ and (b) (Ba_0.90_Eu_0.10_)_2_Ga_8_SiS_16_. Observed intensities (cross), calculated patterns (red line), Bragg positions (tick mark), and difference plot (blue line) are presented.

To investigate the phases of the obtained phosphors and a knowledge of their crystal structure, the Rietveld analysis^22^ was performed using the single crystal structure data of BaGa_2_SiS_6_ (ICSD file no. 184747) and Ba_2_Ga_8_SiS_16_ (ICSD file no. 194875), respectively, as references to approach a dependable approximation of the actual crystal structure. The experimental, calculated, and structural results of the SXRD refinement of (Ba_0.95_Eu_0.05_)Ga_2_SiS_6_ and (Ba_0.90_Eu_0.10_)_2_Ga_8_SiS_16_ are shown in Fig. 1a and b, respectively.

The final refinement converged with weighted-profiles of *R*_p_ = 3.04% and *R*_wp_ = 4.28% of (Ba_0.95_Eu_0.05_)Ga_2_SiS_6_; *R*_p_ = 1.59% and *R*_wp_ = 2.72% of (Ba_0.90_Eu_0.10_)_2_Ga_8_SiS_16_, thus revealing the good quality of the refinement. The crystallographic data are summarized and the selected bond lengths are available in Table 1 and 2, respectively.

Structural parameters of (Ba_0.95_Eu_0.05_)Ga_2_SiS_6_ and (Ba_0.90_Eu_0.10_)_2_Ga_8_SiS_16_ of Rietveld Refinement from SXRD data at room temperature^a^

**Table:** 

Formula	(Ba_0.95_Eu_0.05_)Ga_2_SiS_6_	(Ba_0.90_Eu_0.10_)_2_Ga_8_SiS_16_
Symmetry	Trigonal	Hexagonal
Space group	*R*3 (no. 146)	*P*6_3_*mc* (no. 186)
Lattice parameters	*a* = *b* = 9.55032(28)	*a* = *b* = 10.83380(10)
	*c* = 8.63634(27)	*c* = 11.86626(18)
	*V* = 682.18(4)	*V* = 1206.164(25)
*R*_wp_	4.28%	4.68%
*R*_p_	3.04%	2.75%
*χ* ^2^	3.805	2.873

a Lattice parameters: *a* and *c* in Å, *V* in Å^3^; *T* = 298 K; BL01C2 beamline of NSRRC, *λ* = 0.774908 Å; Site Occupancy Fraction (S.O.F.); *U*_iso_ in Å^2^.

**Table:** 

(Ba_0.95_Eu_0.05_)Ga_2_SiS_6_					
Atom	*x*/*a*	*y*/*b*	*z*/*c*	S.O.F	*U*_iso_*100
Ba	0.00000	0.000000	0.4988(26)	0.9500	6.59
Ga	0.0537(13)	0.8276(10)	0.9498(29)	0.6700	0.30
Si	0.049351	0.7379(22)	0.8921(74)	0.3300	1.34
S(1)	0.8527(13)	0.5886(12)	0.5146(28)	1.0000	3.12
S(2)	0.8136(15)	0.7627(13)	0.8284(27)	1.0000	1.63
Eu	0.000000	0.000000	0.4988(26)	0.0500	6.59

**Table:** 

(Ba_0.90_Eu_0.10_)_2_Ga_8_SiS_16_					
Atom	*x*/*a*	*y*/*b*	*z*/*c*	S.O.F	*U*_iso_*100
Ba(1)	0.333300	0.666700	0.0253(8)	0.9500	4.53
Ba(2)	0.333300	0.666700	0.5156(8)	0.9500	2.59
Ga(1)	0.0048(17)	0.3384(11)	0.3262(65)	0.8600	1.42
Ga(2)	0.8846(5)	0.1154(5)	0.0746(13)	1.0000	2.16
Si	0.0048(17)	0.3384(11)	0.3262(65)	0.1400	1.42
S(1)	0.6625(14)	0.0040(14)	0.0164(7)	1.0000	1.72
S(2)	0.2255(11)	0.7745(11)	0.2637(18)	1.0000	1.06
S(3)	0.5593(9)	0.4407(9)	0.2791(20)	1.0000	1.61
S(4)	0.8977(11)	0.1023(11)	0.2664(18)	1.0000	0.53
S(5)	1.000000	0.000000	−0.0162(22)	1.0000	2.70
Eu(1)	0.333300	0.666700	0.0253(8)	0.0500	4.53
Eu(2)	0.333300	0.666700	0.5156(8)	0.0500	2.59

**Table:** Selected interatomic bond distances^a^ of (Ba_0.95_Eu_0.05_)Ga_2_SiS_6_ and (Ba_0.90_Eu_0.10_)_2_Ga_8_SiS_16_ at room temperature

(Ba_0.95_Eu_0.05_)Ga_2_SiS_6_		(Ba_0.90_Eu_0.10_)_2_Ga_8_SiS_16_	
(Ba/Eu)-S1	3.45104(10) (3×)	(Ba1/Eu1)-S1	3.61231(3) (6×)
(Ba/Eu)-S1^i^	3.50580(8) (3×)	(Ba1/Eu1)-S2	3.47936(4) (3×)
(Ba/Eu)-S2	3.51719(8) (3×)	(Ba1/Eu1)-S3	3.54776(4) (3×)
(Ba/Eu)-S2^i^	3.60984(11) (3×)	(Ba2/Eu2)-S1	3.54578(3) (6×)
Ga-S1	2.27107(7)	(Ba2/Eu1)-S2	3.60961(4) (3×)
Ga-S1^i^	2.40444(7)	(Ba2/Eu1)-S3	3.71969(4) (6×)
Ga-S2	2.20099(6)	(Ga1/Si)-S1	2.25675(3)
Ga-S2^i^	2.12290(5)	(Ga1/Si)-S2	2.19921(2)
		(Ga1/Si)-S3	2.19370(2)
		(Ga1/Si)-S4	2.32944(2)
		Ga2-S1	2.19539(2) (2×)
		Ga2-S4	2.28965(3)
		Ga2-S5	2.41845(2)

a Bond distance in Å.

**Fig. 2:**
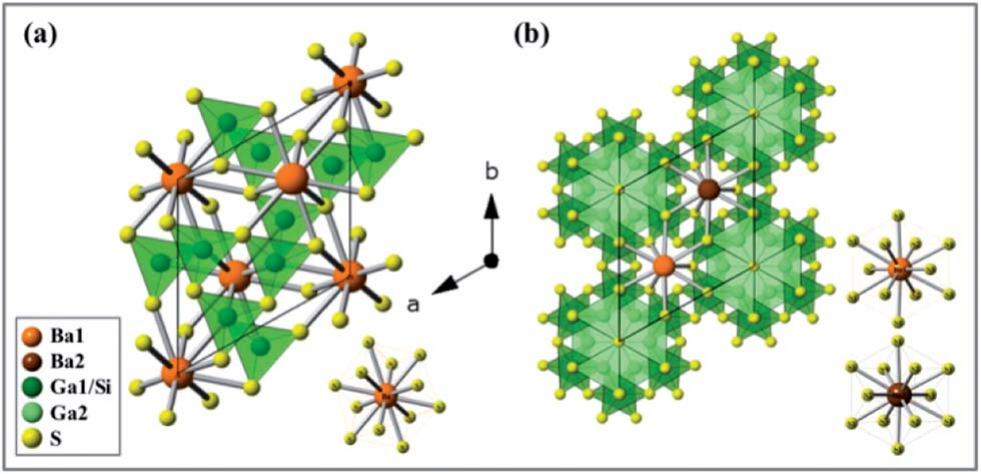
(a) Schematic crystal structure of BaGa_2_SiS_6_ (trigonal, *R*3) (a) and Ba_2_Ga_8_SiS_16_ (hexagonal, *P*6_3_*mc*) (b) viewed down the *c*-axis. Orange, brown, green, light green and yellow sphere balls describe Ba1, Ba2, Ga1/Si, Ga2, S and Si atoms. The insets show the coordination environment around BaS_12_ (left-down) of BaGa_2_SiS_6_, Ba1S_12_ (right-up) and Ba2S_12_ (right-down) of Ba_2_Ga_8_SiS_16_.

The as-synthesized BaGa_2_SiS_6_ phosphor was found to crystallize in the space group *R*3 of the trigonal system, whereas the Ba_2_Ga_8_SiS_16_ in the space group *P*6_3_*mc* of the hexagonal system. Fig. 2a presents the exact crystal structure of BaGa_2_SiS_6_ viewed along the *c*-axis and a single Ba atomic site (BaS_12_). Fig. 2b indicates the crystal structure of Ba_2_Ga_8_SiS_16_, which shows two crystallographically-independent Ba atomic sites (Ba1 and Ba2) along with the Ga/SiS_4_ tetrahedral coordination.

The grain size and morphology of the two phosphor particles characterized by SEM show that the as-synthesized phosphor was composed of irregular granular micro crystals. The nominal stoichiometry was also verified by EDS measurement, as shown in Fig. S1.†

### 3.2 Spectroscopic study of the BaGa_2_SiS_6_:Eu^2+^ and Ba_2_Ga_8_SiS_16_:Eu^2+^ phosphor

**Fig. 3:**
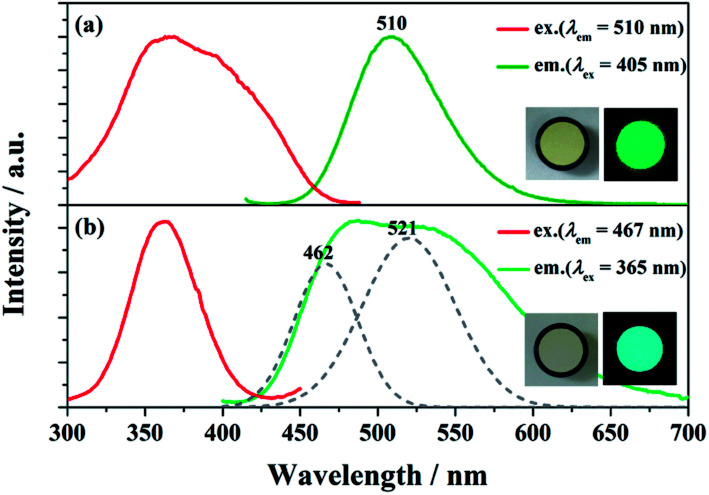
(a) PLE/PL spectra of BaGa_2_SiS_6_:0.01Eu^2+^. (b) PLE/PL spectra and the PL deconvolution (dashed line) of Ba_2_Ga_8_SiS_16_:0.01Eu^2+^. The insets show photos of the phosphor taken under normal light (left) and 365 nm UV light (right).

The PLE and PL spectra of BaGa_2_SiS_6_:0.01Eu^2+^ and Ba_2_Ga_8_SiS_16_:0.01Eu^2+^ is represented in Fig. 3. The BaGa_2_SiS_6_:Eu^2+^ phosphor is excitable around 350 to 450 nm and generates a green emission peaking at 510 nm, and the Ba_2_Ga_8_SiS_16_:Eu^2+^ phosphor exhibits a broadband cyan emission upon excitation at 365 nm. The two emission bands of Ba_2_Ga_8_SiS_16_:Eu^2+^ are attributed to the two sites of barium atom (Ba1 and Ba2) in the Ba_2_Ga_8_SiS_16_ lattice.

**Fig. 4:**
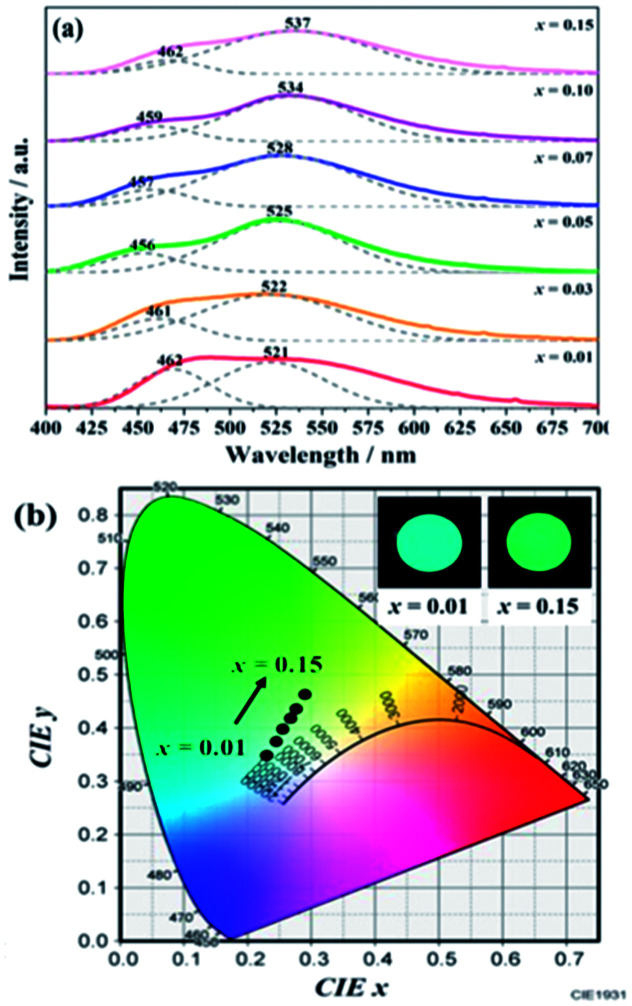
PL spectra (a) and variation in CIE chromaticity coordinates (b) of (Ba_1−*x*_Eu_*x*_)_2_Ga_8_SiS_16_ (0.01 ≤ *x* ≤ 0.15) phosphor as a function of Eu^2+^ content.

As indicted in Fig. 4, the Eu^2+^ emission peak changes from 462 and 521 nm (1% Eu^2+^) to 462 and 537 nm (15% Eu^2+^) with the increasing Eu^2+^ concentration. In addition, the intensity of the short emission decreases while that of the long emission enhances as Eu^2+^ concentration increases. This may result from the fact that Eu^2+^ occupy in different sites, *viz*, Ba1 and Ba2 sites in the Ba_2_Ga_8_SiS_16_ lattice, and the energy transfer between Eu^2+^ ions in the two different sites occur with increasing Eu^2+^ concentration.

**Fig. 5:**
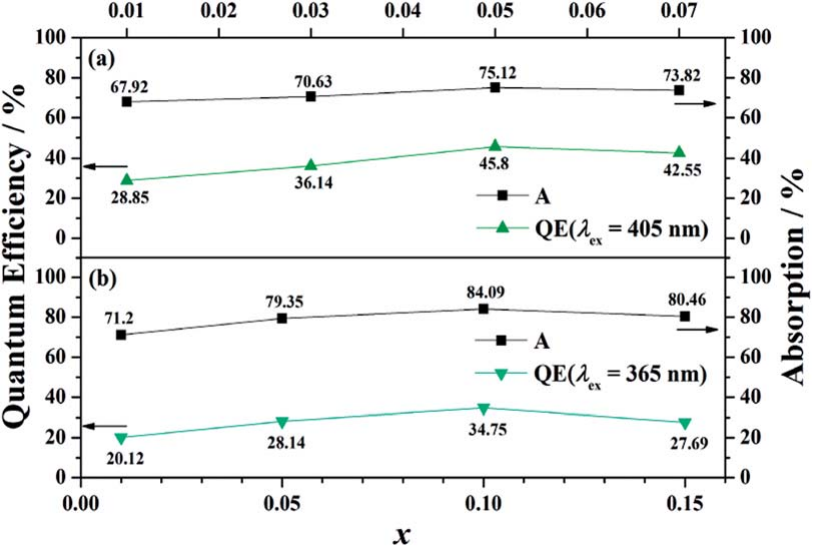
Absorption (A) and external quantum efficiency (EQE) as a function of *x* in (Ba_1−*x*_Eu_*x*_)Ga_2_SiS_6_ (0.01 ≤ *x* ≤ 0.10) and (Ba_1−*x*_Eu_*x*_)_2_Ga_8_SiS_16_ (0.01 ≤ *x* ≤ 0.15) under excitation at 405 and 365 nm, respectively.

Moreover, BaGa_2_SiS_6_:Eu^2+^ exhibits a higher external quantum efficiency (EQE) values than that of Ba_2_Ga_8_SiS_16_:Eu^2+^ (Fig. 5). The comparatively high asymmetry in BaGa_2_SiS_6_:Eu^2+^ crystal may be the major reason for the higher EQE. With an increase in absorption, the EQE value for (Ba_1−*x*_Eu_*x*_)Ga_2_SiS_6_ (0.01 ≤ *x* ≤ 0.10) increases and reaches maximum values of about 45.80% at *x* = 0.10, whereas (Ba_1−*x*_Eu_*x*_)_2_Ga_8_SiS_16_ (0.01 ≤ *x* ≤ 0.15) reaches maximum values of about 34.75% at *x* = 0.10, as shown in Fig. 5.

### 3.3 Time resolved measurement and thermal luminescence properties of the BaGa_2_SiS_6_:Eu^2+^ and Ba_2_Ga_8_SiS_16_:Eu^2+^ phosphor

**Fig. 6:**
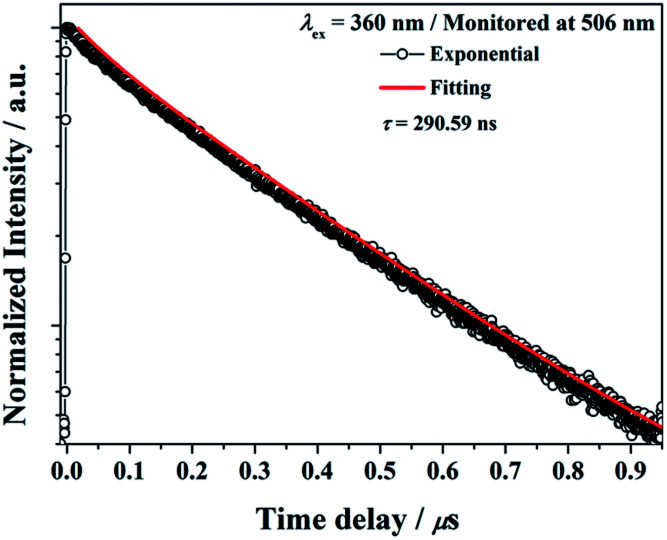
Decay curve for BaGa_2_SiS_6_:Eu^2+^ phosphor (black line) and curve-fitting (red line) under 360 nm excitation and monitored at 506 nm.

The decay curve of BaGa_2_SiS_6_:Eu^2+^ phosphor excited at 360 nm and monitored at 506 nm is presented in Fig. 6. The measured lifetime is related to the first-order exponential equation given by^27^
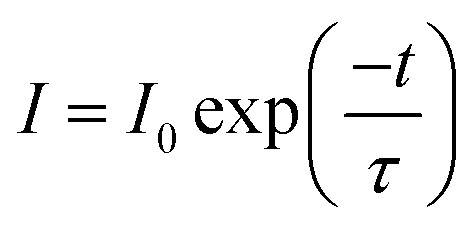


The luminescence decay times *τ* was calculated to be 290.59 ns for BaGa_2_SiS_6_:Eu^2+^, the result is acceptable for the parity-allowed 4f^6^5d^1^ → 4f^7^ transitions of Eu^2+^ and rapid enough for LED lighting applications. Moreover, the well-fitting results by an exponential decay with a single component illustrate that Eu^2+^ ions occupy only one site in the BaGa_2_SiS_6_ host.

**Fig. 7:**
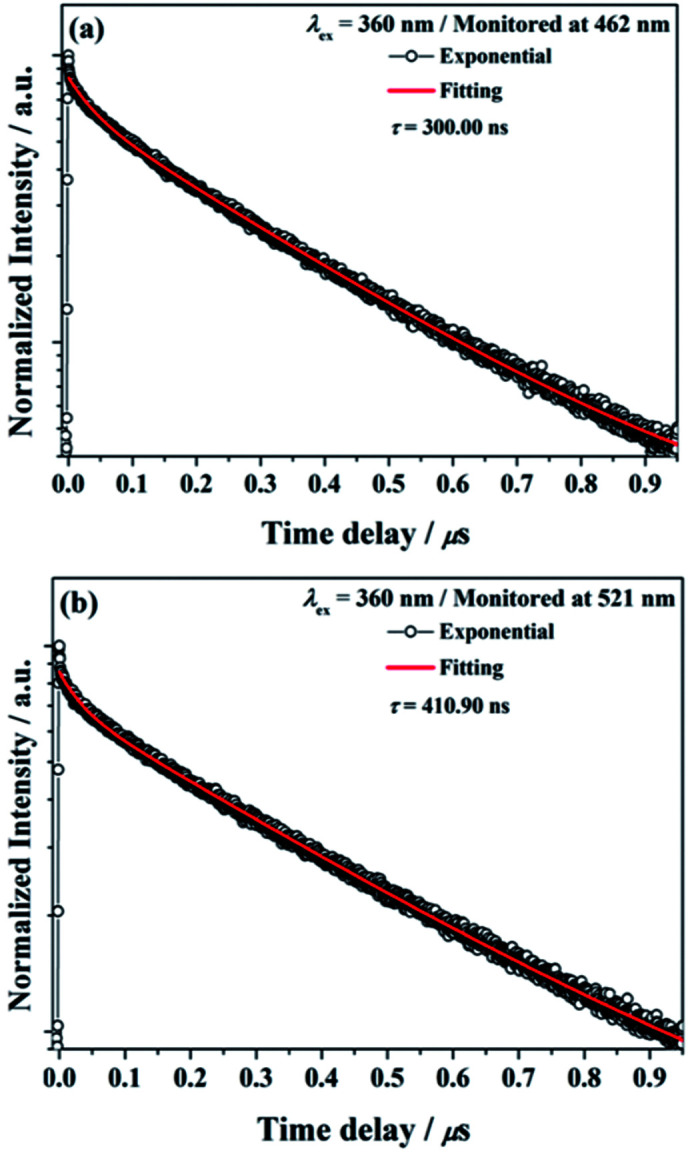
Decay curve for Ba_2_Ga_8_SiS_16_:Eu^2+^ phosphor (black line) and curve-fitting (red line) under 360 nm excitation and monitored at (a) 462 nm and (b) 521 nm.

The decay curves of Ba_2_Ga_8_SiS16:Eu^2+^ phosphor monitored at 462 nm (*τ* = 300.00 ns) and monitored at 521 nm (*τ* = 410.90 ns) under 360 nm excitation are illustrated in Fig. 7a and b, respectively. The results indicated that the Eu^2+^ ions occupied the two different Ba^2+^ ions coordination environment in the Ba_2_Ga_8_SiS_16_ host.

**Fig. 8:**
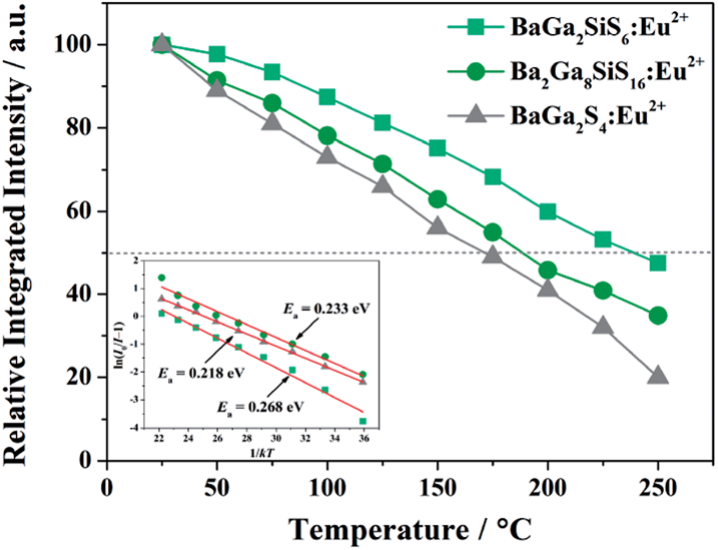
Temperature dependence of relative PL integrated intensity for BaGa_2_SiS_6_:Eu^2+^, Ba_2_Ga_8_SiS_16_:Eu^2+^, and BaGa_2_S_4_:Eu^2+^ over the range 25 to 250 °C. The inset shows the fitted PL integrated intensity and the calculated thermal activation energy (*E*_a_) as a function of temperature.

Both thiosilicate phosphors were found to remain intact when left in the air at ambient temperature for 10–14 days with 60–70% relative humidity. Thermal luminescence quenching property of a phosphor plays an important role for LED applications. Fig. 8 shows temperature dependence of relative PL integrated intensity for BaGa_2_SiS_6_:Eu^2+^, Ba_2_Ga_8_SiS_16_:Eu^2+^, and BaGa_2_S_4_:Eu^2+^ over the range 25 to 250 °C. The thermal stability of the as-prepared thiogallate phosphors is stronger than that of the ternary sulfide BaGa_2_S_4_:Eu^2+^, which perhaps belongs to the stiff (SiS_4_) tetrahedral network that makes the host more stable. The lower left inset of Fig. 8 presents the calculated thermal activation energy (*E*_a_) expressed by the following equation:^28^3
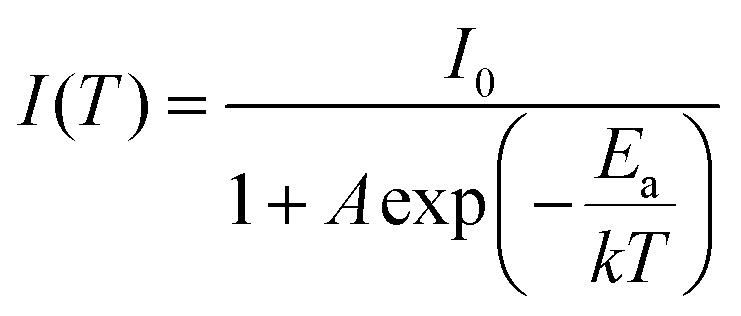
where *I*_0_ and *I*(*T*) represent the PL integrated intensity at room temperature and testing temperature (25–250 °C), respectively, and *k* the Boltzmann constant. The values of *E*_a_ for BaGa_2_SiS_6_:Eu^2+^, Ba_2_Ga_8_SiS_16_:Eu^2+^, and BaGa_2_S_4_:Eu^2+^ were estimated to be 0.268, 0.233 and 0.218 eV, respectively.

### 3.4 CIE chromaticity coordinates and performance of LED devices based on BaGa_2_SiS_6_:Eu^2+^ phosphor

**Fig. 9:**
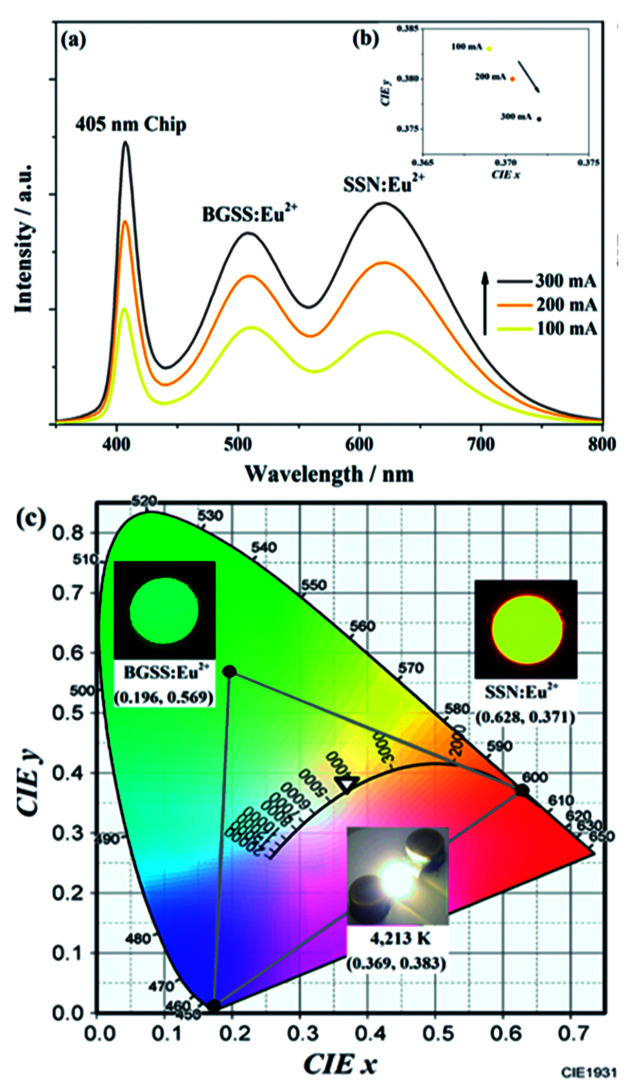
(a) EL spectra of the device using 405 nm LED chip with green-emitting BaGa_2_SiS_6_:Eu^2+^ phosphor, and red-emitting Sr_2_Si_5_N_8_:Eu^2+^ phosphor and (b) variation in CIE chromaticity coordinates of the WLED operated under different currents (100 to 300 mA). (c) CIE chromaticity coordinates of the used phosphors and the fabricated LED are presented. The insets show the used phosphors and the LED device photos recorded under 365 nm excitation.

To reveal the prospective use of BaGa_2_SiS_6_:Eu^2+^ for PC-WLED application, the BaGa_2_SiS_6_:0.01Eu^2+^ phosphor was utilized to fabricate a WLED device driven by 100 to 300 mA current with red-emitting Sr_2_Si_5_N_8_:Eu^2+^ and a 405 nm LED chip shown in Fig. 9.

The EL intensity of the blue, green, and red bands of the white LED device increased while increasing the forward-biased current from 100 to 300 mA, and the saturation phenomenon was not observed even at a high forward current of 300 mA, as illustrated in Fig. 9a. As shown in Fig. 9b, with an increase in the driving current, the CIE chromaticity coordinates shifted slightly. The results demonstrated the excellent colour stability of the BaGa_2_SiS_6_:0.01Eu^2+^ phosphor. In Fig. 9c the studies indicate that this novel green phosphor is a potential candidate for white LED, especially for the generation of warm white light with an optimum CRI of 88 and a CCT value of 4213 K.

## 4. Conclusions

In summary, we have investigated two new Eu^2+^-doped BaGa_2_SiS_6_ and Ba_2_Ga_8_SiS_16_ thiogallate phosphors. The crystal structure and luminescence performance of both the phosphors were studies in detail. The results reveal that the green-emitting Eu^2+^-doped BaGa_2_SiS_6_ and the colour-tunable Eu^2+^-doped Ba_2_Ga_8_SiS_16_ could be excited over a broad range of wavelength thus generating a broadband emission. The green-emitting Eu^2+^-doped BaGa_2_SiS_6_ phosphor was integrated together with a red-emitting phosphor and a blue chip to obtain a warm-white-light LED device an optimum CRI value of 88 and CCT value of 4213 K. Our investigation indicates the potential use of this phosphor in phosphor-converted LED for the lighting application.

## Conflicts of interest

There are no conflicts of interest to declare.

## Supplementary Material

RA-008-C8RA01113C-s001
