# Supplementary material for: Novel Eu2+-activated thiogallate phosphors for white LED applications: structural and spectroscopic analysis
Source: RSC Adv. 2018 Mar 27;8(21):11725–30. doi: 10.1039/c8ra01113c (PMC9079137; doi:10.1039/c8ra01113c)
Supplement: RA-008-C8RA01113C-s001 [file RA-008-C8RA01113C-s001.pdf]

Electronic Supplementary Material (ESI) for RSC Advances.
This journal is © The Royal Society of Chemistry 2018

Novel Eu^{2+}-activated Thiogallate Phosphors for White LED Applications: Structural and Spectroscopic Analysis

Szu-Ping Lee[†], *Ting-Shan Chan*[‡], *Somrita Dutta*[†] *and Teng-Ming Chen*[*,†]

[†]Phosphors Research Laboratory, Department of Applied Chemistry and Institute of Molecular Science, National Chiao Tung University, Hsinchu 30010, Taiwan.
[‡]National Synchrotron Radiation Research Center, Hsinchu 30076, Taiwan

Supporting Information.

The following files are free of charge.

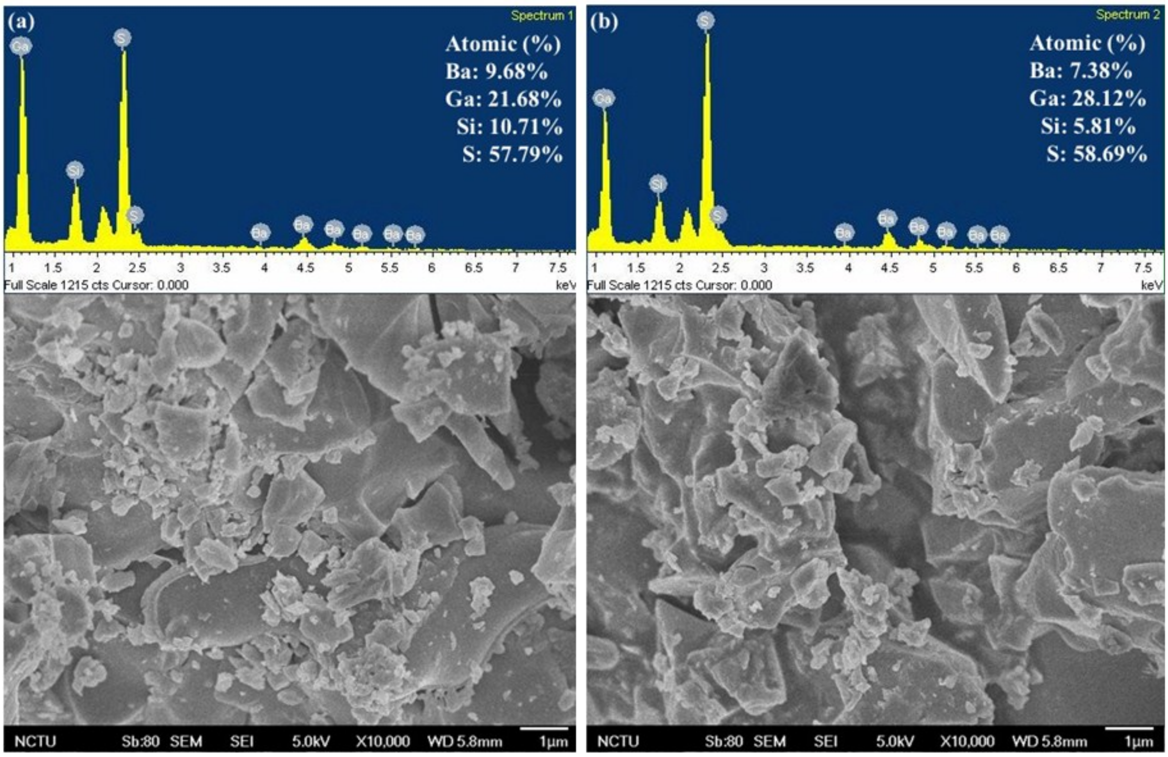

Figure S1. EDS spectra and SEM image of as-synthesizes (a) $BaGa_2SiS_6$ and (b) $Ba_2Ga_8SiS_{16}$ phosphor.
